# Indo-Pacific humpback dolphins (*Sousa chinensis*) in Hong Kong: Modelling demographic parameters with mark-recapture techniques

**DOI:** 10.1371/journal.pone.0174029

**Published:** 2017-03-29

**Authors:** Stephen C. Y. Chan, Leszek Karczmarski

**Affiliations:** The Swire Institute of Marine Science and School of Biological Sciences, Faculty of Science, The University of Hong Kong, Cape d’Aguilar, Shek O, Hong Kong; Institute of Deep-sea Science and Engineering, Chinese Academy of Sciences, CHINA

## Abstract

Indo-Pacific humpback dolphins (*Sousa chinensis*) inhabiting Hong Kong waters are thought to be among the world's most anthropogenically impacted coastal delphinids. We have conducted a 5-year (2010–2014) photo-ID study and performed the first in this region comprehensive mark-recapture analysis applying a suite of open population models and robust design models. Cormack-Jolly-Seber (CJS) models suggested a significant transient effect and seasonal variation in apparent survival probabilities as result of a fluid movement beyond the study area. Given the spatial restrictions of our study, limited by an administrative border, if emigration was to be considered negligible the estimated survival rate of adults was 0.980. Super-population estimates indicated that at least 368 dolphins used Hong Kong waters as part of their range. Closed robust design models suggested an influx of dolphins from winter to summer and increased site fidelity in summer; and outflux, although less prominent, during summer-winter intervals. Abundance estimates in summer (N = 144–231) were higher than that in winter (N = 87–111), corresponding to the availability of prey resources which in Hong Kong waters peaks during summer months. We point out that the current population monitoring strategy used by the Hong Kong authorities is ill-suited for a timely detection of a population change and should be revised.

## Introduction

Biological management of species and population has to begin with accurate estimates of population parameters [1]. The knowledge of species-specific life-history traits and reliable estimates of population parameters, size and structure are not only instrumental in the understanding of the dynamics of natural populations [2], but a *must be* for the design and implementation of effective management strategies [1,3]. Policymakers depend on such data for their management decisions and, inevitably, the effectiveness of management policies depends on the robustness of the scientific evidence from the field. Incomplete data, inaccurate estimates or simply a "sloppy science" can lead to misguided policies which in turn can hamper the conservation and management efforts [3–5]. However, obtaining accurate estimates is often challenging, especially for large and agile marine animals such as cetaceans that travel over large distances and remain submerged for considerable periods of time, which often makes observational sampling impossible. For such species, photo-identification (photo-ID) of individuals, using natural marks on their body (e.g. scars, pigmentation pattern, etc.) provides a minimally intrusive and effective method of collecting photo-capture-recapture data for subsequent mark-recapture analyses [6]. A number of key demographic parameters can be estimated from mark-recapture studies, all of which provide input parameters that can be used further in constructing population management models to advise conservation measures [7–11]. Although in that respect many cetacean studies lag behind similar studies of terrestrial species, recent advances in field research design and analytical techniques have set a stage for considerable advances in the detail, quality and accuracy of cetacean mark-recapture studies [12–15], which have greatly benefitted the work presented here.

The Indo-Pacific humpback dolphin (*Sousa chinensis*), locally in China and Taiwan known as Chinese White Dolphin (hereafter referred to as humpback dolphin), inhabits shallow coastal waters of the eastern Indian Ocean and western Pacific [16,17]. Their inshore distribution and narrow habitat selectivity, typical for all species of the genus *Sousa* [18–20], frequently places them on a collision course with a wide range of anthropogenic activities. Their preferred inshore habitats are often in close proximity to areas of intense coastal fisheries and aquaculture and, especially in southeast Asia, urban and industrial developments that are increasingly affected by pollutions, heavy sea traffic, and various degrees of habitat destruction [21–26]; all of which may contribute to population decline [21,22,27–29].

In China, where the species' past distribution may have been continuous along the southeast coast [30–32], there are now only few remnant populations along the 1500 km of coast from Sanniang Bay, Beibu Gulf in the southwest to Ningde in the northeast, and across the Taiwan Strait along a narrow coastal strip off Taiwan's west coast [33,34]. The population numbers across the region are thought to be low, ranging from few tens to low few hundred [31,35,36], only exceptionally reaching over a thousand individuals [37]. Humpback dolphins inhabiting coastal waters of the Pearl River Estuary (PRE) are thought to number *ca*. 2,500 individuals [38] (although this estimate has to be viewed cautiously [25]), which makes them the largest population of this species along the Chinese coast [39], possibly the largest in the whole of southeast Asia. This population, however, is under a tremendous anthropogenic pressure. It has been suggested that few, if any, other dolphin populations known to science face the range and intensity of threats that occur in the PRE [25,40]. Recent demographic analyses [27] indicate a population decline of 2.46% per annum, suggesting that it may diminish by 74% within the lifespan of three generations.

In Hong Kong waters, the easternmost reaches of the PRE, humpback dolphins have attracted public, scientific and conservation attention since the mid-1990s [41–44], instigated at first by large-scale anthropogenic impacts resulting from a massive construction of the new Hong Kong International Airport. However, despite the previous efforts, considerable information gaps remain and much of the population vital parameters and structure are still poorly understood. Mark-recapture estimates of the dolphin numbers in Hong Kong waters were first attempted in early 2000s [45], along with early cross-border surveys in the mainland part of PRE [46], but the results remained preliminary and never published. The dynamics of dolphin movement, emigration-immigration patterns, and robust estimates of population parameters have never been quantitatively addressed. All current conservation management decisions pertaining to humpback dolphins in Hong Kong are based on annual monitoring programme (line transect surveys) conducted under the auspices of the Agriculture, Fisheries and Conservation Department (AFCD) of the Hong Kong Government [43,47,48], but results of these surveys have to be viewed cautiously due to numerous conceptual and methodological deficiencies which have left many questions unanswered (for a detailed critique see Wilson et al [40]). Thus, there was a need for a study that would make use of rigorously applied quantitative techniques and deliver a reliable measure of the population vital parameters pertaining to humpback dolphins in Hong Kong waters, an issue never properly addressed and long overdue in both Hong Kong and the PRE.

The study reported here represents a first comprehensive mark-recapture analysis of humpback dolphins in Hong Kong waters and quantifies demographic parameters of a population that is open to recruitment and removal between sampling occasions [49]. With Cormack-Jolly-Seber (CJS) models, we estimate survival rate [50] and quantify the presence and proportion of temporary visitors (referred to as transient individuals) [51]. We use POPAN parameterization of Jolly-Seber model [52] to estimate super-population size, the total number of individuals that use Hong Kong waters as part of their range. We apply the robust design models [53–56], an approach which combines the concepts of open and closed population into a single model with two levels of sampling units, to determine temporary emigration rates, heterogeneity in capture probabilities, effects of season and seasonal abundance. Our estimates of population parameters, size and seasonal dynamics emphasise the intricate connectivity of the dolphins seen in Hong Kong waters with humpback dolphins elsewhere in the PRE. We highlight the importance of a rigorous scientific protocol in gaining the understanding of the population processes that ultimately determine the species biological persistence. As conservation and management efforts are generally only as effective as how accurate is the scientific evidence that guides them, we trust that the data presented here will benefit informed management decisions pertinent to the conservation of humpback dolphins in Hong Kong and across the PRE, serving as a model, both applicable and achievable elsewhere in the region.

## Methods

### Study area

**Fig 1:**
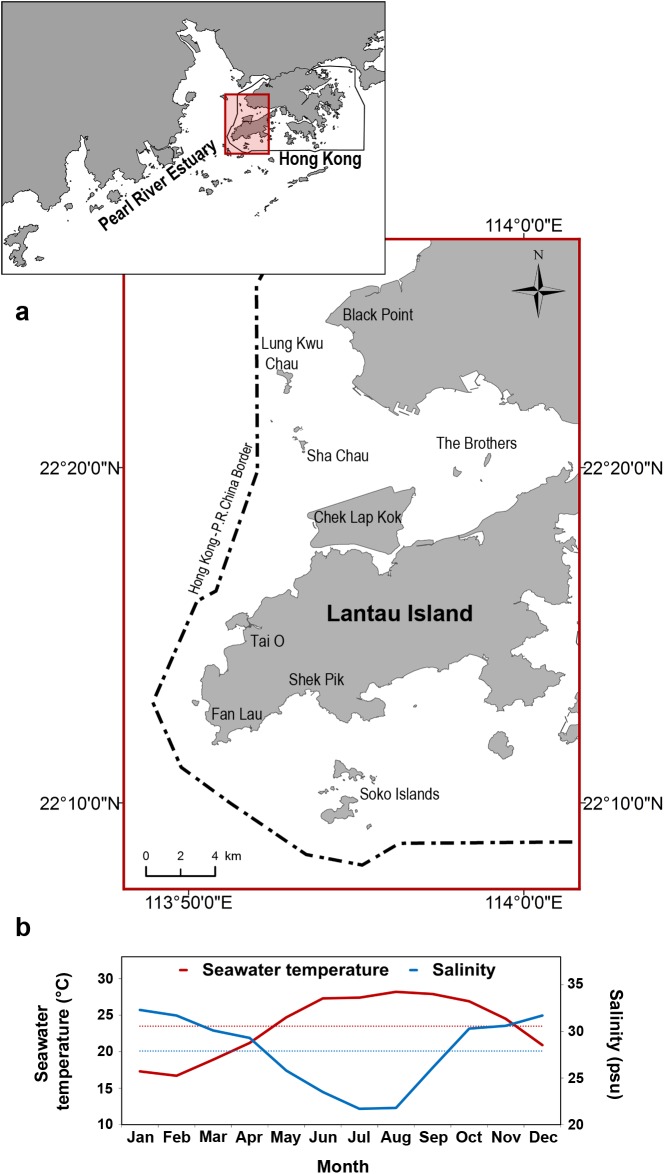
(a) The study area in western Hong Kong waters, at the eastern reaches of Pearl River Estuary, southeast China. Photo-ID surveys covered the entire region of Hong Kong territorial waters known to be inhabited by Indo-Pacific humpback dolphins [43], including west coast of Lantau Island to the extent of Sha Chau-Lung Kwu Chau Marine Park and Black Point in the north, The Brothers islands in the north-east, Shek Pik peninsula and Soko Islands in the south/south-east. The dolphins are rarely seen further east in Hong Kong waters [43,48] The thick broken line represents the administrative border separating Hong Kong Special Administrative Region (Hong Kong S.A.R.) and People's Republic of China, which demarcates the western boundary of the study area. The map was generated by S.C.Y. Chan using software ArcGIS (Version 10.2, http://www.esri.com/software/arcgis). (b) The mean monthly (solid lines) and annual mean (dotted horizontal lines) seawater temperature and salinity, averaged across measurements at the surface, mid-water column and at the bottom, in western Hong Kong waters during 2010–2013. (Data source: Environmental Protection Department HKSAR, 2015; http://epic.epd.gov.hk/EPICRIVER/marine/).

Hong Kong is located at the easternmost reaches of the Pearl River Estuary (PRE), one of the largest estuarine systems in southeast China (Fig 1A). The area is influenced by subtropical monsoon and experience seasonal fluctuations in seawater temperature, rainfall, river discharge and primary productivity, all of which increase during summer months. Water salinity on the other hand, increases during winter, when the freshwater discharge of Pearl River decreases [57–59]. The estuarine influence and seasonal fluctuations due to the Pearl River discharge are most pronounced in the west of Hong Kong and gradually decrease to the east [57–60]. Western Hong Kong waters, with depth generally < 20 m, are thought to represent the easternmost boundary of the PRE population of humpback dolphins, as the animals are rarely seen anywhere further east in the PRE region [43].

### Definitions

In this study, the term "population" refers to the dolphins that used the study area as part of their home range during the study period; it does not refer to genetic or geographic isolation unless specifically stated. Each time a dolphin or group of dolphins were seen and photo-ID data were collected is referred to as a "dolphin encounter". The term "sighting" refers to a case when at least one ID-image of an individual was taken and met the minimum quality criteria for the full suite of photo-ID analyses described below.

Two annual seasons were distinguished, "summer" and "winter", based on mean seawater temperature (similar as in Karczmarski et al [61] and Chang et al [62]) and salinity in western Hong Kong waters, both averaged across measurements at the sea surface, mid-water and at the bottom over years (Fig 1B). The consecutive months with the mean monthly seawater temperature higher than the annual mean of 23.5°C and mean monthly salinity lower than the annual mean of 27.9psu (Fig 1B) were defined as "summer". The period when mean monthly seawater temperature dropped below and salinity rose above the annual mean was termed “winter”. During October and November, however, the mean monthly seawater temperature is that of summer and water salinity is that of winter; hence this 2-month period was split half and equally assigned to the summer (October) and winter (November). Consequently, the period of May—October is referred hereafter as "summer" and the period of November—April as "winter". This definition of seasons, which is alike that applied in recent studies off Taiwan's west coast [62] and corresponds to seasonal dynamics described in several environmental studies in Hong Kong and PRE [58,59,63], corresponds also with the pattern of our survey intensity due to sea conditions (Table 1) and thus it facilitates the structuring of our mark-recapture dataset for examining the effects of field effort and season.

**Table 1:** Survey effort, shown per summer/winter season, and summary of photo-ID data (Q≥80) obtained in western Hong Kong waters during May 2010—October 2014.

Season	Period	No. of Months	No. of Surveys	No. of Hours	No. of Catalogued IDs		
					Non-calf (D ≥ 1)	Adult + Subadult (D ≥ 1)	Highly marked Adult + Subadult (D ≥ 3)
2010 Summer	2010 May– 2010 Oct	6	29	77	163	148	129
2011 Winter	2010 Nov– 2011 Apr	6	12	50	47	45	40
2011 Summer	2011 May– 2011 Oct	6	45	204.5	181	163	145
2012 Winter	2011 Nov– 2012 Apr	6	13	54	110	99	86
2012 Summer	2012 May– 2012 Oct	6	50	220.5	207	191	165
2013 Winter	2012 Nov– 2013 Apr	6	14	64.5	116	105	91
2013 Summer	2013 May– 2013 Oct	6	48	251.5	245	221	195
2014 Winter	2013 Nov– 2014 Apr	5	33	152.5	171	149	137
2014 Summer	2014 May– 2014 Oct	6	42	250	281	258	230
Overall		53	286	1324.5	380	346	305

Four age-classes were distinguished based on their external appearance, coloration [41,44] and body size: calf, juvenile, subadult, and adult (see also Karczmarski [64] and Chang et al [62] for comparison). Calves are dark grey to light grey in colour, two-third or less the length of an adult and regularly accompanied by an adult. Individuals approximately 2m long, light grey at the dorsal body with or without dark spots, visibly less robust than adults, which often swam independently, were classified as juveniles. Adults are at least 2.5m in length, with robust bodies and well-developed dorsal ridge, entirely pink in colour or mostly pink with dark spots. Dolphins with the external appearance in between the age-class of juvenile and adult, with body length and robustness similar to that of adult but still with greyish cast in their colouration and/or dense dark spots on the upper body were classified as subadults.

### Field data collection

From May 2010 to October 2014, photo-identification (photo-ID) surveys were conducted using an 8-m boat powered by one 140HP 4-stroke outboard engine. The surveys were undertaken year-round, weather permitting, with sea state ≤ 3 in Beaufort scale, following similar procedures as described by Karczmarski et al [65] and whenever possible (weather permitting) they covered the entire study area. Once a dolphin or group of dolphins was sighted, the animals were approached at low speeds (< 6 km/h) and group size, age-class composition and behavioural state were recorded. Subsequently, the dolphins were photographed by at least two researchers, aiming at close-ups of their dorsal fins and upper bodies, using digital cameras Canon EOS 1D Mark III and/or Mark IV equipped with 100-400mm f/4.5–5.6 zoom lenses, with a conscious effort to capture both left and right body sides of all group members regardless of distinctiveness, age-class or behaviour of the individuals.

### Photographic identification

All collected digital images were processed and catalogued using the photo-ID data management software system DISCOVERY [66]. Image quality (Q) of all ID-photographs and individual distinctiveness (D) of all photo-captured individuals were independently assessed and verified by at least two experienced researchers who jointly managed the photo-ID database. Image quality was graded on a scale Q 1–100 [65] according to the exposure, focus, parallax, and whether the entire dorsal fin was visible above the water. Individual distinctiveness was rated on a scale D 0–5 [67] according to notches on a dorsal fin and pigmentation of a dorsal ridge. All photo-ID records and associated data were archived in the DISCOVERY database; however, to avoid any potential misidentification of individuals and to minimize unequal catchability related biases, only high-quality images (Q≥80) of highly distinctive individuals (D≥3) were used in mark-recapture analyses described further. As the majority of calves and juveniles were poorly marked, to avoid biases these age-classes were not included in further analyses.

Following the above mentioned assessments, a mark-ID ratio ($\hat{\theta }$) was estimated to represent the proportion of highly distinctive individuals that could be reliably and repeatedly identified. The ratio was calculated for all encounters in which all group members were photo-captured with high-quality photographs:

$$\hat{\theta }=\frac{Number\;of\;high-quality\;images\;(Q\geq 80)\;of\;highly\;marked\;individuals\;(D\geq 3)}{Number\;of\;high-quality\;images\;(Q\geq 80)\;of\;all\;individuals}$$

Subsequently, the mean and standard error were calculated across all encounters.

### Population site fidelity

Individual site fidelity was measured by calculating lagged identification rates (LIR), which represent the probability that an individual identified at any particular time will be identified again in the study area certain time units later [68]. Movement models were fitted to the observed data with the application of software program SOCPROG 2.5 [69], and Akaike Information Criterion (AIC) and quasi-likelihood AIC (QAIC) were used to select the best fitted model. Bootstrap method was used to estimate standard error and 95% confidence interval of the observed data and the movement models [70].

### Mark-recapture analyses

The sighting histories of highly distinctive subadults and adults were analysed using program MARK [71]. We applied a suite of open population models and robust design models similarly as described by Silva et al [12]. Cormack-Jolly-Seber (CJS) model [72–74] was used to estimate apparent survival rates (ϕ) and recapture probabilities (p) [50], and POPAN model [52] was used to provide super-population estimates (N). Closed robust design model [53–56] was applied to estimate temporary emigration parameters (γ” and γ’; see explanation below) and seasonal abundance (N_i_).

In open population models (CJS and POPAN models), each season was considered as one sampling occasion with dolphin sightings pooled together for every summer and winter separately (in all subsequent modelling, the seasonal survival probabilities were converted to represent annual rate). Goodness-of-fit (GOF) tests were performed to identify the most parameterized starting (general) CJS model that adequately fit the data. Program U-CARE [75] was used to test for the significance of unequal survival and recapture probabilities among individuals. Variance inflation factor ($\hat{c}$), representing the degree of overdispersion of data, was estimated by bootstrap GOF and median-$\hat{c}$ methods implemented in program MARK. Candidate models, including the general model and more reduced models, were built with various effects on survival and capture probabilities: time dependent (‘t’), constant over occasions (‘.’), seasonal variation (‘season’), annual variation (‘yr’), survey intensity (‘effort’), cohort effect (‘cohort’), transient effect with two time-since-marking (TSM) classes (heterogeneity in apparent survival rate; ‘a2’) and trap-dependence effect with two TSM classes (heterogeneity in capture rate; ‘*m’). Selection of the best candidate models was achieved by comparing the corrected Akaike Information Criterion (AIC_c_), or quasi-likelihood AIC_c_ (QAIC_c_) if overdispersion was significant (when $\hat{c}$ > 1); models with ΔAIC (or ΔQAIC) > 10 received no support [76]. Further restriction to models with ΔAIC (or ΔQAIC) < 5 (P.S. Hammond, University of St Andrews, UK; personal. comm.) did not generate notably different results and therefore the approach of Burnham and Anderson [76] was followed. To test the significance of certain model effect, likelihood ratio test (LRT) was performed between a pair of nested models, which consists of a more general model and its reduced model. Model averaging across the candidate model set by AIC_c_ weight (or QAIC_c_ weight) was used to calculate the weighted averages of estimated parameters.

Closed robust design models assume that the population is open to recruitment and removal between primary periods (primary capture occasions) while closed within each primary period. The sighting history dataset used in open models were subsampled to fulfil this assumption. A 2-month period representing the peak of each season (August-September for “Summer” and February-March for “Winter”) was used as a primary period, and each 2-week within the primary period was pooled as a secondary occasion so that the sampling period was sufficiently short compared to the time interval between the primary capture occasions. In addition to the effects on survival and capture probabilities, various temporary emigration models were developed, including “no movement” (γ” = 0 and γ’ = 1), Markovian movement (γ”≠ γ’), random movement (γ” = γ’) and “even-flow” (γ” = 1-γ’) models, where γ” is the probability of temporary emigration outside the study area given that the individual was present in the previous primary period, and γ’ is the probability of staying outside the study area given that the individual was not present in the previous primary period. No GOF tests are available for robust design models, so the variance inflation factor was adopted from CJS models following a conservative approach, even though lower level of overdispersion was expected from the subsampled dataset.

### Total population size

The population size estimates from mark-recapture models refer only to the population of highly marked individuals. As our field and lab photo-ID protocol ensured that, at each encounter, all marked and unmarked individuals had the same probability of being captured, the total population size (${\hat{N}}_{T}$) was projected using mark-ID ratio as follows:

$${\hat{N}}_{T}=\hat{N}/\hat{\theta }$$

where $\hat{N}$ is the mark-recapture population estimate and $\hat{\theta }$ is the mark-ID ratio. The variance of total population size was estimated following Urian et al [77] as:

$$var({\hat{N}}_{T})={{\hat{N}}_{T}}^{2}\left(\frac{var(\hat{N})}{{\hat{N}}^{2}}+\frac{var(\hat{\theta })}{{\hat{\theta }}^{2}}\right)$$

The lower and upper log-normal 95% confidence intervals were calculated by ${\hat{N}}_{T}^{lower}={\hat{N}}_{T}/C$ and ${\hat{N}}_{T}^{Upper}={\hat{N}}_{T}\times C$, where [78]

$$C=\exp\left(1.96\sqrt{\ln\left(1+\frac{var({\hat{N}}_{T})}{{{\hat{N}}_{T}}^{2}}\right)}\right).$$

### Ethics statement

The study followed internationally accepted photo-ID field protocol and no permits were required.

## Results

### Survey effort and database

During the continuous 4.5-year study period, 286 surveys were conducted across all months but one, November 2013, due to unfavourable weather conditions (Table 1). Survey effort was higher in summer, as sea conditions in Hong Kong worsen considerably during winter months. Over 350,000 ID-images were taken, of which > 53,000 met the quality criteria Q ≥ 80. A total of 380 distinctive (D≥1) non-calf individuals were identified and catalogued (Q≥80), of which 305 adult and subadult dolphins were sufficiently distinctive (D≥3) for mark-recapture analyses.

### Discovery rate and site fidelity

**Fig 2:**
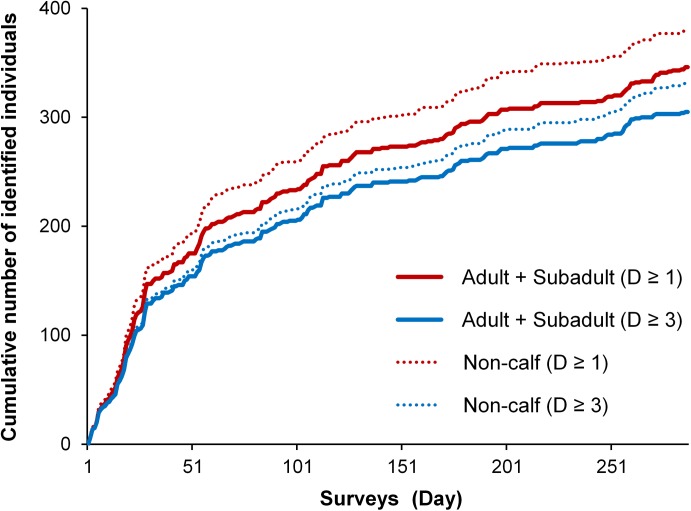
Discovery curves showing the cumulative number of humpback dolphins photo-captured during 286 photo-ID surveys conducted in western Hong Kong waters between May 2010 and October 2014. All identified individuals (D≥1): red lines; all highly distinctive individuals (D≥3): blue lines; adults and subadults (used in mark-recapture analyses described further): solid lines; all individuals excluding calves (non-calf individuals): dotted lines.

**Fig 3:**
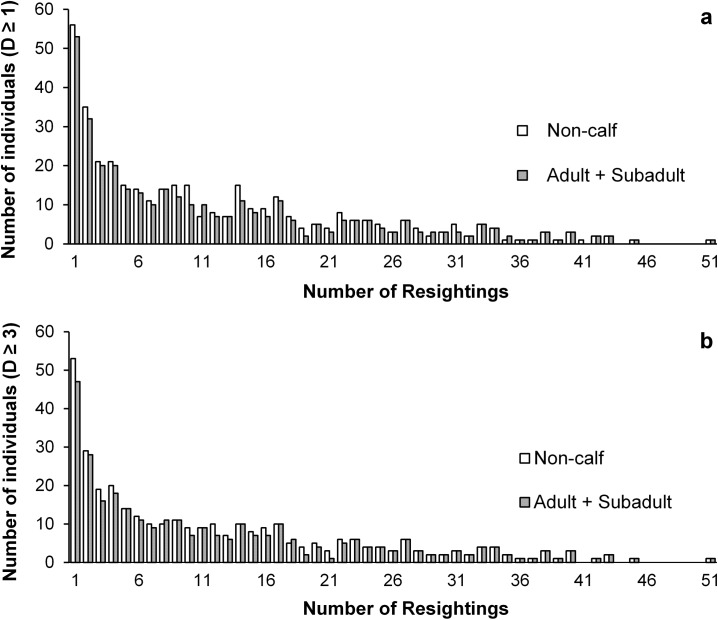
**Sighting frequency distribution of (a) all identified individuals (D**≥**1) and (b) all highly distinctive individuals (D**≥**3) of humpback dolphins seen in western Hong Kong waters between May 2010 and October 2014.** Adults and subadults (used in mark-recapture analyses described further): grey bars; all individuals excluding calves (non-calf individuals): white bars.

Although the cumulative number of photographically identified individuals continued to increase throughout the study period, the rate of discovering new individuals gradually slowed down after the initial 30 survey days (Fig 2). Individual sighting rates were generally low (Fig 3), with approximately one-sixth of all identified dolphins seen only once. However, 25.3% (n = 96) of all catalogued individuals (D≥ 1) and 25.2% (n = 77) of highly distinctive individuals (D≥ 3) were seen ≥ 18 times (on average once every three months). The most frequently sighted individual was photo-captured 51 times.

**Fig 4:**
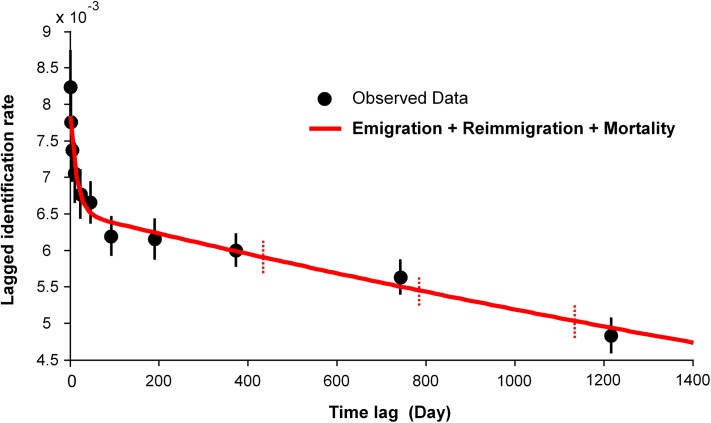
Lagged identification rates of highly marked (D≥3) adult and subadult humpback dolphins in western Hong Kong waters and best supported model “*Emigration + Reimmigration + Mortality*” (variance inflation factor = 1.2402). Bootstrap error bars of observed data and the movement model with 5000 replications are shown as solid error bars and dotted error bars, respectively.

Lagged identification rates (LIR) of highly marked (D≥3) adult and subadult dolphins declined sharply from time lag of day-1 to day-24, after which the rate of decline slowed down, but continued to drop throughout the 1,400 day-lag projection (Fig 4). The movement model that best fit the observed pattern was “Emigration + Reimmigration + Mortality” (Fig 4). The next candidate models, “Emigration / Mortality” and “Closed: Emigration + Reimmigration” received no model support (ΔQAIC > 20) and both failed to address the initial rapid decline of LIR.

### Open population models

GOF TEST 3 and TEST 2 with program U-CARE indicated significant transient effect on survival probability (χ^2^ = 85.81, df = 14, P < 0.001) and trap-dependence effect on capture probability (χ^2^ = 67.78, df = 13, P < 0.001), respectively. The variance inflation factor ($\hat{c}$) of the general model including both transient and trap-dependent effects was 1.516 in median-$\hat{c}$ and 1.895 in bootstrap GOF tests. Following a conservative approach, the highest value ($\hat{c}$ = 1.895) was used to correct the degree of overdispersion in QAIC_c_ model selection.

Among 26 CJS candidate models (Table 2), the full time-dependent model (Model 17) displayed a poor fit to the data. The incorporation of transient effect to survival rate (‘a2’) (Model 15; ΔQAIC_c_ = 4.56) and heterogeneity in recapture probability (‘*m’) (Model 12; ΔQAIC_c_ = 6.05) considerably improved the model fit. The results of LRT confirmed the significance of the two factors (Model 15 vs. 17: χ^2^ = 19.119, df = 7, P = 0.008; and Model 12 vs. 17: χ^2^ = 20.608, df = 7, P = 0.004). The effects of cohort (‘cohort’) (Models 9 and 25) and survey intensity (‘effort’) (Models 16, 18 and 20) had little bearing in explaining the data. In overall, there was considerable model uncertainty, with 11 models of ΔQAIC_c_<10, integrating the mixture of constant (‘.’), seasonal (‘season’), annual ('yr') and cohort effects on TSM survival rate, and time-dependent (‘t’) and seasonal recapture probabilities with and without heterogeneity.

**Table 2:** Cormack-Jolly-Seber (CJS) candidate models arranged in ascending order of quasi-likelihood Akaike Information Criterion (QAIC_c_). Survival probability: ϕ; recapture probability: p; variance inflation factor: $\hat{c}$ = 1.895. Refer to the Methods section for the modelling notation of various effects.

#	Model	QAIC_c_	ΔQAIC_c_	QAIC_c_ Weights	Model Likelihood	No. of Parameters	QDeviance
1	ϕ(a2-./season) p(t*m)	1050.74	0.00	0.435	1.000	18	222.87
2	ϕ(a2-season/season) p(t*m)	1052.82	2.07	0.154	0.355	19	222.87
3	ϕ(a2-season/season) p(t)	1052.93	2.18	0.146	0.336	12	237.44
4	ϕ(a2-./.) p(t*m)	1053.03	2.29	0.139	0.318	17	227.24
5	ϕ(a2-season/.) p(t*m)	1055.08	4.33	0.050	0.115	18	227.21
6	ϕ(a2-season/.) p(t)	1055.16	4.41	0.048	0.110	11	241.72
7	ϕ(season) p(t)	1058.93	8.18	0.007	0.017	10	247.54
8	ϕ(a2-yr/yr) p(t*m)	1059.70	8.95	0.005	0.011	20	227.66
9	ϕ(a2-cohort/cohort) p(t)	1060.27	9.53	0.004	0.009	20	228.24
10	ϕ(.) p(t)	1060.35	9.60	0.004	0.008	9	251.00
11	ϕ(a2-season/season) p(season*m)	1060.36	9.62	0.004	0.008	7	255.08
12	ϕ(t) p(t*m)	1061.91	11.17	0.002	0.004	22	225.70
13	ϕ(a2-season/season) p(.*m)	1062.40	11.65	0.001	0.003	8	255.08
14	ϕ(a2-t/t) p(t*m)	1063.36	12.61	0.001	0.002	28	214.50
15	ϕ(a2-t/t) p(t)	1063.40	12.66	0.001	0.002	22	227.19
16	ϕ(a2-season/season) p(effort*m)	1066.58	15.83	<0.001	<0.001	7	261.30
17	ϕ(t) p(t)	1067.97	17.22	<0.001	<0.001	15	246.31
18	ϕ(a2-season/season) p(effort)	1083.37	32.63	0	0	6	280.12
19	ϕ(a2-season/season) p(season)	1084.66	33.92	0	0	5	283.44
20	ϕ(t) p(effort)	1095.19	44.45	0	0	9	285.84
21	ϕ(a2-season/season) p(yr)	1130.20	79.46	0	0	8	322.89
22	ϕ(t) p(.)	1188.40	137.66	0	0	4	389.20
23	ϕ(a2-season/season) p(.)	1188.52	137.78	0	0	5	387.29
24	ϕ(a2-./.) p(.)	1190.67	139.92	0	0	3	393.48
25	ϕ(a2-season/season) p(cohort)	1192.70	141.96	0	0	11	379.27
26	ϕ(.) p(.)	1201.15	150.40	0	0	2	405.97

**Fig 5:**
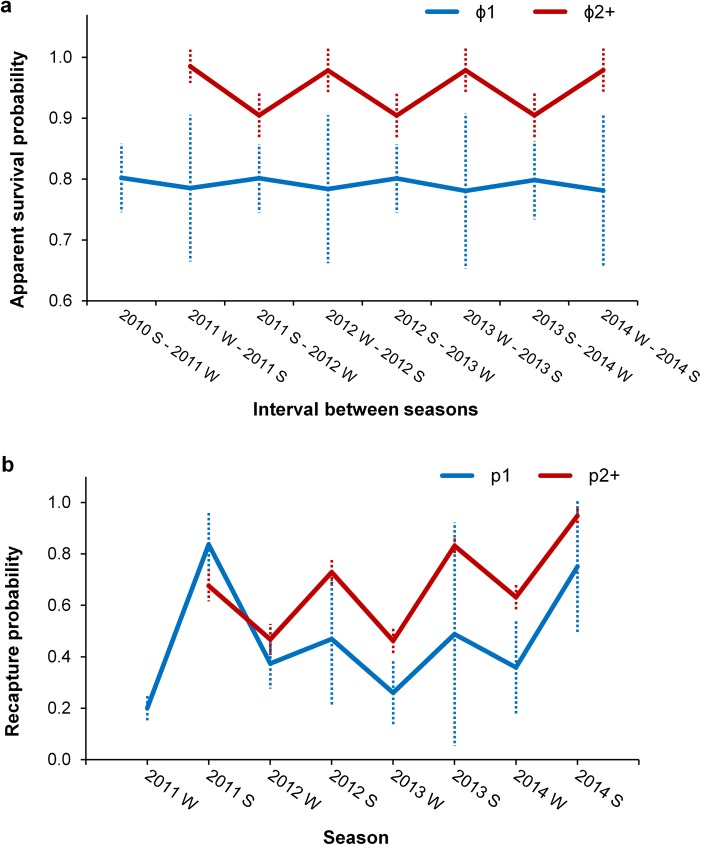
**Weighted averages of (a) apparent survival probability estimates (ϕ) and (b) recapture probability estimates (p) over 17 best candidate Cormack-Jolly-Seber (CJS) models with one standard error as error bars (dotted lines).** ϕ_1_/p_1_ (blue lines) and ϕ_2+_/p_2+_ (red lines) represent the probabilities of 1^st^ TSM class and 2^nd^ TSM class individuals, respectively. ‘S’ and ‘W’ denotes summer and winter, respectively. The first and second capture probability estimates (winter and summer 2011) should be viewed cautiously, given the lower survey intensity (Table 1) due to logistic challenges of a newly initiated study.

Weighted averages of estimated CJS parameters are shown in Fig 5. Apparent survival probabilities of previously marked individuals (2^nd^ TSM class) varied with season and were higher during winter-summer intervals (${\bar{\phi }}_{2+,w-s}$ = 0.980 ± SE 0.034) than summer-winter intervals (${\bar{\phi }}_{2+,s-w}$ = 0.905 ± SE 0.038). Newly marked animals (1^st^ TSM class) had lower apparent survival rate (${\bar{\phi }}_{1}$ = 0.792 ± SE 0.097) which varied only slightly between seasons (Fig 5A). There were differences in recapture probabilities between first (p_1_; 1^st^ TSM class) and repeatedly recaptured (p_2+_; 2^nd^ TSM class) individuals, but the standard errors of p_1_ were large. In overall, recapture rates were higher during summer (${\bar{p}}_{1,s}$ = 0.469 to 0.837; ${\bar{p}}_{2+,s}$ = 0.676 to 0.948) than winter months (${\bar{p}}_{1,w}$ = 0.201 to 0.374; ${\bar{p}}_{2+,w}$ = 0.461 to 0.631) (Fig 5B).

In POPAN open models, TSM models are not applicable, which reduced the number of potential candidate models to 17. The best fit models (ΔQAIC_c_<10; Table 3, models 1–7) had either seasonal or constant survival probability, but all had time-dependent capture rates. Models accounting for survey effort (Model 1) and effects of season (Model 2) on entrance probability better explained the observed pattern than constant or time varying effects models (Model 5 and Model 7, respectively).

**Table 3:** Jolly-Seber (JS) candidate models with POPAN parameterization arranged in ascending order of quasi-likelihood Akaike Information Criterion (QAIC_c_). Survival probability: ϕ; capture probability: p; probability of entering super-population: pent; variance inflation factor: $\hat{c}$ = 1.895. Refer to the Methods section for the modelling notation of various effects.

#	Model	QAICc	ΔQAICc	QAICc Weights	Model Likelihood	No. of Parameters	QDeviance
1	ϕ(season) p(t) pent(effort)	1089.64	0.00	0.387	1.000	14	-291.85
2	ϕ(season) p(t) pent(season)	1090.79	1.16	0.217	0.561	14	-290.70
3	ϕ(.) p(t) pent(effort)	1090.93	1.29	0.204	0.526	13	-288.52
4	ϕ(.) p(t) pent(season)	1092.19	2.55	0.108	0.279	13	-287.26
5	ϕ(season) p(t) pent(.)	1093.78	4.14	0.049	0.126	13	-285.66
6	ϕ(.) p(t) pent(.)	1094.89	5.25	0.028	0.072	12	-282.51
7	ϕ(season) p(t) pent(t)	1098.67	9.03	0.004	0.011	19	-293.10
8	ϕ(.) p(t) pent(t)	1100.17	10.53	0.002	0.005	18	-289.55
9	ϕ(t) p(t) pent(.)	1102.54	12.90	0.001	0.002	18	-287.17
10	ϕ(t) p(t) pent(t)	1107.89	18.25	<0.001	<0.001	24	-294.26
11	ϕ(season) p(season) pent(.)	1125.93	36.29	0	0	6	-239.29
12	ϕ(season) p(effort) pent(.)	1126.99	37.35	0	0	6	-238.22
13	ϕ(season) p(season) pent(t)	1131.74	42.10	0	0	13	-247.71
14	ϕ(.) p(.) pent(t)	1259.99	170.35	0	0	11	-115.37
15	ϕ(t) p(.) pent(.)	1275.50	185.86	0	0	11	-99.86
16	ϕ(t) p(.) pent(t)	1277.32	187.69	0	0	18	-112.39
17	ϕ(.) p(.) pent(.)	1280.12	190.48	0	0	4	-81.06

After correction for the mark-ID ratio ($\hat{\theta }$), the total super-population size (${\hat{N}}_{T}$) was estimated at 368 individuals (CV = 0.071; 95% CI = 320–422) (Table 4). Seasonal population size estimates (${\hat{N}}_{iT}$) ranged from 222 (CV = 0.108; 95% CI = 179–274) to 285 individuals (CV = 0.085; 95% CI = 242–337) with a consistent pattern of fluctuation between seasons.

**Table 4:** Weighted average estimates of super-population size ($\hat{N}$) and seasonal population size (${\hat{N}}_{i}$) over the best 10 Jolly-Seber (JS) candidate models with POPAN parameterization, and total population size (${\hat{N}}_{T}$ and ${\hat{N}}_{iT}$) corrected with mark-ID ratio $\hat{\theta }$) for the proportion of highly-marked individuals. Coefficient of variation: CV; 95% log-normal confidence intervals: 95% CI.

Season	Mark-ID ratio		Super-population size estimates				
	$\hat{\theta }$	SE	$\hat{N}$	CV	${\hat{N}}_{T}$	CV	95% CI
Overall	0.855	0.059	314	0.016	368	0.071	320–422
			Seasonal population size estimates				
			${\hat{N}}_{i}$	CV	${\hat{N}}_{iT}$	CV	95% CI
2010 Summer			198	0.089	231	0.113	185–288
2011 Winter			189	0.084	222	0.108	179–274
2011 Summer			208	0.059	243	0.091	204–290
2012 Winter			199	0.063	233	0.094	194–279
2012 Summer			224	0.042	262	0.081	224–307
2013 Winter			214	0.048	250	0.084	212–295
2013 Summer			236	0.043	276	0.081	235–323
2014 Winter			229	0.051	268	0.086	227–317
2014 Summer			244	0.050	285	0.085	242–337

### Robust design models

With no GOF tests available, the starting closed robust design model included TSM survival probability and heterogeneous capture rates following the CJS models (Table 5, Model 15). However, the difference between capture and recapture rates (p ≠ c; Models 12 and 15) and individual heterogeneity in capture probabilities (π≠ 0; Model 7, 8, 10, 15) were not supported in the model selection. Time variation effect (‘t’) on capture probabilities was applied as it was strongly supported in open models. Constant over time and seasonal effects on survival probability, with and without TSM variation, were supported by the four best fit models (ΔQAIC<10). Among temporary emigration patterns tested, both “no movement” (γ" = 0 γ' = 1; Model 9) and “even-flow” models (γ" = 1-γ'; Model 11) had a poor fit to the data (Table 5). Markovian movement models (γ"≠γ'; Models 1, 2 and 3) were more parsimonious than Random movement models (γ” = γ’; Model 4), which was further supported by LRT results (e.g. Model 1 vs 4; χ^2^ = 8.965, df = 2, P = 0.0113). For either of these two movement patterns, seasonal effect on temporary emigration probabilities dominated the top four models.

**Table 5:** Closed robust design candidate models arranged in ascending order of Quasi Akaike Information Criterion (QAIC_c_). Survival probability: S; emigration probabilities: γ" and γ'; probability of heterogeneity mixture: π; capture probability: p; recapture probability: c; variance inflation factor: $\hat{c}$ = 1.895. Refer to the Methods section for the modelling notation of various effects.

#	Model	QAIC_c_	ΔQAIC_c_	QAIC_c_ Weights	Model Likelihood	No. of Parameters	QDeviance
1	S(a2-./.) γ"(season) γ'(season) π = 0 p = c(t)	-664.48	0.00	0.644	1.000	46	1070.62
2	S(.) γ"(season) γ'(season) π = 0 p = c(t)	-662.35	2.13	0.222	0.345	45	1074.91
3	S(a2-season/season) γ"(season) γ'(season) π = 0 p = c(t)	-660.07	4.41	0.071	0.110	48	1070.71
4	S(a2-./.) γ" = γ'(season) π = 0 p = c(t)	-659.83	4.65	0.063	0.098	44	1079.59
5	S(a2-./.) γ"(.) γ'(.) π = 0 p = c(t)	-645.20	19.28	<0.001	<0.001	44	1094.22
6	S(a2-./.) γ" = γ'(.) π = 0 p = c(t)	-639.18	25.30	0	0	43	1102.39
7	S(a2-./.) γ"(season) γ'(season) π(.) p = c(t)	-636.93	27.55	0	0	78	1027.15
8	S(a2-./.) γ"(season) γ'(season) π(season) p = c(t)	-636.38	28.10	0	0	79	1025.42
9	S(a2-./.) γ" = 0 γ' = 1 π = 0 p = c(t)	-628.88	35.60	0	0	42	1114.83
10	S(a2-./.) γ"(season) γ'(season) π(t) p = c(t)	-626.81	37.67	0	0	86	1018.89
11	S(a2-./.) γ" = 1-γ'(season) π = 0 p = c(t)	-625.54	38.94	0	0	46	1109.56
12	S(a2-./.) γ"(season) γ'(season) π = 0 p(t) c(t)	-580.17	84.31	0	0	59	1126.54
13	S(a2-./.) γ"(season) γ'(season) π = 0 p(.) c(.)	-505.83	158.65	0	0	33	1257.05
14	S(a2-./.) γ"(season) γ'(season) π = 0 p = c(.)	-493.43	171.05	0	0	25	1286.24
15	S(a2-t/t) γ"(t) γ'(t) π(t) p(t) c(t)	-466.64	197.84	0	0	134	1063.19

During the summer-winter intervals, the emigration probability (${\bar{\gamma "}}_{s-w}$ = 0.490 ± SE 0.063) was considerably higher than the probability of re-immigration (1- ${\bar{\gamma ’}}_{s-w}$ = 0.123 ± SE 0.208). During the winter-summer intervals the opposite was true; the emigration rate (${\bar{\gamma "}}_{w-s}$) dropped to 0.010 ± SE 0.052 and the rate of return (1- ${\bar{\gamma ’}}_{w-s}$) increased to 0.757 ± SE 0.083.

Seasonal abundance estimates, after correction for the mark-ID ratio ($\hat{\theta }$), ranged from 87 to 111 in winter and 144 to 231 in summer (Table 6). The abundance estimates of the first two field seasons (summer 2010 and winter 2011; Table 6) were deemed unreliable due to large CV and wide 95% CI.

**Table 6:** Weighted average estimates of seasonal abundance (${\hat{N}}_{i}$) over the best five closed robust design candidate models, total abundance estimates (${\hat{N}}_{iT}$) corrected for the proportion of highly-marked individuals (mark-ID ratio $\hat{\theta }$ = 0.855) and the number of surveys of the subsampled dataset. Coefficient of variation: CV; 95% log-normal confidence intervals: 95% CI. There were considerable uncertainties in the estimates in summer 2010 and winter 2011, and these should be viewed with caution.

Season	Number of Surveys	Seasonal abundance estimates				
		${\hat{N}}_{i}$	CV	${\hat{N}}_{iT}$	CV	95% CI
2010 Summer	15	255	0.334	298	0.341	156–571
2011 Winter	4	48	0.420	56	0.425	25–124
2011 Summer	24	123	0.099	144	0.121	114–182
2012 Winter	4	75	0.318	87	0.326	47–162
2012 Summer	23	150	0.094	175	0.117	139–220
2013 Winter	5	84	0.196	98	0.208	65–146
2013 Summer	20	183	0.057	214	0.089	180–255
2014 Winter	14	95	0.074	111	0.101	91–135
2014 Summer	20	198	0.039	231	0.079	198–270

## Discussion

Although our study had to be limited to the administrative border of Hong Kong territorial waters, there were no geographic barriers to the studied animals, and humpback dolphins were frequently seen travelling across the border into the territorial waters of the P.R. China. Demographic closure was therefore not expected. Seasonality turned out to be a prominent feature. The CJS mark-recapture models indicate a significant transient effect and seasonal variation in apparent survival probabilities, an obvious result of a fluid movement ranging well beyond our restricted study area. In Hong Kong, the survival rate of adults approximates 0.98, while super-population size estimates with POPAN models indicate that at least 368 dolphins rely on Hong Kong costal habitats as part of their home range. Temporary emigration parameters indicate an influx of dolphins during the transition periods from winter to summer and increased site fidelity in summer; and outflux, although less prominent, during the summer-winter intervals. Correspondingly, seasonal abundance estimates generated with robust design models were larger in summer (N = 144–231) than winter (N = 87–111). These seasonal fluctuations match well with prey availability, which in Hong Kong waters peaks during summer months (see further).

### Assumption validation

While performing a suite of mark-recapture analysis of the Indo-Pacific humpback dolphins in Hong Kong waters, several standard open model assumptions were made: (1) no marks are lost or missed, (2) all samples are instantaneous and released immediately after sampling, (3) all marked animals have the same survival probability from one sampling occasion to the next, and (4) at a given sampling occasion, all marked animals have the same capture probability. Violations of the first two assumptions are considered negligible as notches on dorsal fins are long-lasting and only top-quality ID-images were used in our study. Furthermore, photo-ID sampling, with no physical captures, is both non-invasive and instantaneous.

However, given the differences in the behaviour of individuals and their ability to travel outside the limited (restricted by the administrative border) study area, variability of apparent survival rate and capture probability were inevitable, violating assumptions 3 and 4. To minimize the biases arising from individual heterogeneity, a conscious effort was made to equalise catchability during field surveys, with sampling effort similarly distributed across all animals, marked and unmarked individuals, at each encounter. Any further violation of equal capture probabilities were minimized by rigorous application of image-quality and individual distinctiveness criteria. As the results of GOF test indicated that certain violation of these assumptions was still present, the lack of fit due to such violation was effectively accounted for by stratifying the data structure with TSM models and the resulting overdispersion of data was acceptable ($\hat{c}$<3).

In addition to these standard assumptions, closed robust design models assume closure within each primary sampling period, as compared to the intervals in between the primary periods. Although the dolphins can leave or enter the study area within the 2-month primary sampling periods, the closure assumption could be relaxed if the movement during the periods is completely random or all individuals are present in either the first or last secondary occasion [79]. With only two to four secondary occasions in each primary period, the subsampled sighting history dataset should therefore give relatively unbiased estimations of capture probability and abundance.

### Site fidelity, capture rate and seasonal effects

Recapture probabilities of the 2^nd^ TSM class were higher than that of the 1^st^ TSM class, although SE of the estimates for the 1^st^ TSM class (individuals resighted first time) were large (Fig 5B). The higher recapture probabilities of repeatedly seen individuals (2^nd^ TSM class) may reflect higher site fidelity of some individuals (see also further) which, although frequently travel in and out of Hong Kong territorial waters, use this area more consistently across longer period of time than most other individuals, as suggested also by the wide distribution range of individual sighting frequencies (Fig 3).

Although effects of season and survey effort were not supported in the CJS model selection as explanation of the seasonal fluctuation, it is likely that recapture probabilities were related to survey intensity which was affected by seasonal weather and sea conditions, thus leading to consistent differences in capture rates between seasons (Fig 5B). On the other hand, estimates for the consecutive summers and winters (especially summers) gradually increased across the study period (with the exception of winter and summer 2011; Fig 5B). The survey effort, however, did not increase across years (Table 1), except for winter 2014 which had unusually favourable sea conditions. The increasing trend in recapture probabilities may therefore reflect an improvement of surveying techniques through practice, including photographic and boat manoeuvring skills and perhaps increased skills in detecting individual dolphins out at sea. The first and second capture probability estimates, however, winter and summer 2011, have to be viewed with caution as they are likely artefacts of lower survey intensity and logistic challenges of a newly initiated study.

The pattern of lagged identification rates (LIR) suggests generally low site fidelity to Hong Kong waters. However, following an initial rapid decline of LIR (Fig 4), indicative of no site fidelity across days or weeks, the subsequent considerable change in the rate of decline suggests that across longer time periods (months/years) some individuals display a certain degree of affinity to Hong Kong waters, with a fluid pattern of emigration and re-immigration. This concurs with the pattern of individual sighting frequencies (Fig 3) and with the estimates generated by robust design models which indicate considerable seasonality in movement. During summer-winter intervals, temporary emigration rates exceeded re-immigration, while the opposite was true for the time periods leading from winter to summer, when the influx of animals from other areas was 6-fold that of summer-winter periods. This seasonality is further supported by our estimates of abundance and survival (discussed further). The modelled LIR showed no sign of levelling off, however, which could be due to mortality and permanent emigration, as suggested also by the best-fit movement model. Together with the non-asymptotic discovery curve and low resighting rates, this is indicative of the demographic openness of this population.

Recent study by Or [80] suggests that foraging represents a key determinant of humpback dolphin distribution in Hong Kong waters, while considerable body of evidence assembled by several other studies [81–85] indicates that the seasonal pattern of movement and fluctuations in dolphin abundance correspond to the availability of their prey resources. Stomach content analyses by Barros et al [85] and a recent study by W. Lin of Sun Yat-sen University (unpublished) identified the predominant prey species of humpback dolphins in the PRE region, of which the majority peaks in abundance in Hong Kong waters during summer months (S1 Table). High prey availability provides foraging opportunities, lowers food competition and increases seasonal habitat capacity [86–88], thus likely attracting the dolphins to Hong Kong waters during the transition from winter to summer.

Various degrees of seasonal movement and varying residency have been observed in other populations of the genus *Sousa* [61,89–93] as well as other coastal delphinids [94–100] and the extent of seasonal dynamics generally corresponds to the extent of seasonal climatological fluctuations of the environment. For example, off Zanzibar and northeast Australia [101,102], the effects of season are far less obvious and humpback dolphins appear considerably more resident than they are off southeast South Africa, where coastal prey resources vary considerably across the year (reviewed in Karczmarski [103]) and so does the pattern of occurrence, ranging and group formation displayed by humpback dolphins [61,64,89] as well as sympatrically occurring Indo-Pacific bottlenose dolphins (*Tursiops aduncus*) [104]. Consequently, foraging needs and the annual pattern of resource availability appear to be the primary determinants of site fidelity and movement of coastal dolphins, following the spatio-temporal shifts of their food availability. Clearly, the PRE humpback dolphins are no different.

Although the movement of humpback dolphins outside Hong Kong waters remains little known, our current findings may indicate a larger spatio-temporal distribution pattern in the greater PRE coastal system. With Hong Kong located at the easternmost reaches of PRE and the furthest away from the river mouths (Fig 1), the strong seasonal influx of humpback dolphins at the onset of summer and moderate level of temporary emigration at the beginning of winter may be a reflection of a seasonal movement away from the river mouths in summer and towards the inner estuary during dry winter. Similar pattern has been observed and monitored with acoustic techniques in Xin Huwei River estuary off Taiwan's west coast [105], and concurred with the seasonal fluctuations in the volume of freshwater discharge and prey availability. However, given the considerably greater size and complexity of the PRE coastal system compared to that of Xin Huwei River estuary, further work cross-matching the individual photo-IDs collected across PRE is needed to verify this hypothesis. Remote tracking with the application of satellite-linked transmitters could offer an effective alternative approach and should be encouraged. Such data would be imperative for better understanding of the population processes, especially in the face of currently ongoing and planned large-scale infrastructure projects, such as the Hong Kong–Zhuhai–Macau (HKZM) Bridge [106] and the Third Runway System (3RS) at Hong Kong International Airport [107], which continue to alter (through land reclamation) and degrade dolphin habitat in Hong Kong and the PRE [25]. At present, however, research applications of satellite-linked transmitters in the PRE region do not seem possible due to socio-political constraints.

### Survival rate and movement pattern

Estimates of apparent survival rate represent the true survival of animals and their permanent emigration [50,51]. In long-living mammals such as humpback dolphins, true survival rates of grown individuals are unlikely to change much due to aging in a relatively short period of time. Without any catastrophic events affecting their survival during the study period, individual heterogeneity and seasonal variations of adult survival estimates are therefore expected to be closely related to patterns of emigration [12,51].

In this study, survival heterogeneity by TSM effect was evident from the results of GOF test and CJS model selection. The 2-class TSM structure classified the animals seen once only as “transients” and those seen twice or more as “residents”; but this classification reflects the frequency of occurrence in Hong Kong waters, not their migratory pattern; e.g. none of the “residents” is likely to remain in Hong Kong waters year-round but rather frequently move in and out of the study area (emigrating and re-immigrating). The apparent survival rate of the first TSM class, a mixture of "transient" and "resident" animals, was substantially lower due to permanent emigration of animals seen only once. The proportion of "transient" individuals in the population (T) can be estimated as follows:

$$T=1-(\phi_{T+R}/\phi_{R})$$

where *ϕ*_*T*+*R*_ represents the apparent survival probability of the "transients" and "residents" combined, and *ϕ*_*R*_ represents that of "residents" only [51]. Ranging from 12.5% in summer to 19.3% in winter, the mean proportion of "transients" was estimated at 16.5%, which is almost identical with the percentage of highly distinctive dolphins photo-captured only once (56/346 = 16.2%).

With "transient" individuals excluded from further estimates, seasonal variation of CJS apparent survival rates followed closely the movement patterns generated by the robust design models. The mean summer-winter survival probability (${\bar{\phi }}_{s-w}$ = 0.905) was lower than that for winter-summer intervals (${\bar{\phi }}_{w-s}$ = 0.980; Fig 5A) due to a considerable probability of emigration of even the more resident individuals (${\bar{\gamma "}}_{s-w}$ = 0.490); while the dolphins present in Hong Kong waters during winter were likely to remain there though summer (1- ${\bar{\gamma "}}_{w-s}$ = 0.990) and their survival estimate was the least influenced by transience and emigration. Consequently, the winter-summer estimate of apparent survival rate of the "resident" individuals represents the closest approximation of the true survival rate of humpback dolphins in Hong Kong waters (${\bar{\phi }}_{w-s}$ = 0.980 ± SE 0.034). This estimate is surprisingly high and may have been overestimated due to the potential removal of true mortality together with the "transient" individuals in the TSM models. The extent of such potential bias, however, is likely much less compared to the inevitable serious underestimation if the strong evidence of emigration would not be accounted for. Moreover, the estimate of 0.980 falls within the range of age-specific survival rates of adults inferred from the previously published life-history parameters [44] and is comparable to the only other non-calf survival rate estimate for this species reported for the Eastern Taiwan Strait population [108]; although the latter has to be viewed cautiously, as pointed out in recent critiques [28,62].

On the other hand, although non-calf survival rates can be correlated to the rate of population change and used to estimate the risk of population extinction, our estimated survival rate cannot be directly compared with the threshold values reported recently for the PRE population [109] (*ϕ* = 0.955) and Eastern Taiwan Strait [28] (*ϕ* = 0.963) as those estimates considered all individuals above the age of 1-year and included animals that due to their young age and individual indistinctiveness could not be included in the mark-recapture study reported here. Therefore, the survival rate of 0.980, albeit seemingly high, does not suggest stationary or increasing population (for details see Karczmarski et al [109]) and does not contradicts the declining trend reported by Huang et al [27]. Instead, it indirectly supports the previously voiced concerns that for populations experiencing a multitude of anthropogenic stressors, such as the PRE population [25], low calf survivorship, which inevitably reduces the population recruitment rate, may represent an important factor contributing to the population decline [25,26,62,110], as reported also for other mammals, both marine and terrestrial [111–116]. Neonatal mortality in the PRE is reported to be high [44,117] and might be further exacerbated by infanticidal tendencies of some males [118] and should therefore be a cause for concern; especially when annual recruitment nears or drops below annual removal. Management measures that could facilitate higher calf survivorship could be an important step towards better conservation of the PRE population.

### Population estimates and management implications

The super-population size estimate represents the total number of dolphins that have used Hong Kong waters as part of their range over the study period, while abundance estimates from robust design models indicate the number of dolphins that occur in Hong Kong waters over shorter time-intervals representative of summer and winter. Given the non-asymptotic discovery curve (Fig 2) and dynamic movements in and out of the study area, it could be anticipated that the total POPAN size estimate will gradually increase with longer study period, and our findings confirm this pattern. Seasonal super-population estimates differed only slightly between summer and winter seasons, correspondingly with the seasonality of movement, implying a relatively stable usage of Hong Kong waters by a comparable number of different individuals year-round (Table 4). Although individual differences in the pattern of habitat use can be expected (e.g. “resident” animals use the area more frequently than “transients”), the POPAN estimates across seasons indicate an overall long-term reliance of the dolphins on Hong Kong waters. This is not surprising, given that this area harbours some of the most important foraging grounds in the whole of Eastern PRE [80].

The seasonal difference in abundance was more prominent in the robust design model estimates (Table 6), which were not subject to bias due to individual heterogeneity in capture rates (probability of heterogeneity mixture π = 0 in all supported models; Table 5). This seasonal fluctuation and higher estimates in summer corresponds with seasonal fluctuation in prey availability [81–84] and the seasonal pattern of dolphin movement (discussed earlier). The effect of survey intensity, although cannot be completely ruled out, appears to be negligible; e.g. comparable survey effort in summer and winter 2014 yielded 2-fold difference in abundance estimates, while three times greater number of surveys in winter 2014 compared to any other winter period did not produce a similar increase in the abundance estimate (Table 6). The overall higher estimates in the last four seasons, however, may be related to local management decisions, as in December 2012 the Hong Kong authorities introduced a complete ban of commercial trawling in Hong Kong waters. Given the sudden termination of the fishing pressure of *ca*. 400 trawlers operating in Hong Kong waters [119] with an annual catch of ~12,000 tonnes [120], against a backdrop of ongoing intense trawling in the remaining part of the PRE, it seems possible that the local abundance of dolphin prey species may have increased after December 2012 and such an environmental change may have attracted a larger number of dolphins into Hong Kong waters as soon as the following summer. An ongoing post-ban monitoring project has shown early indications of increase in abundance and biomass of crustaceans and demersal fish as early as the end of 2013 [121]. Consequently, the variation in the abundance estimates in the last four seasons of our study may be indicative of varying and gradually increasing habitat capacity.

The mark-recapture abundance estimates produced in this study are considerably higher than the annual estimates from line-transect surveys conducted by the local authorities during the same period: 75 in 2010, 78 in 2011, 61 in 2012, 62 in 2013, and 61 in 2014 [47,48]. There are likely several reasons for these considerable differences. The two research methods, line-transect and mark-recapture techniques, address a different albeit mutually relevant research question, and therefore the estimates they generate have different meanings, each with their own merits. While the line-transect approach estimates the number of animals that are present in the study area at a given time; the mark-recapture analyses estimate the overall number of animals that use Hong Kong waters at any given time during the study period, including individuals that may not have been physically present in the study area at the specific time of a particular survey. Consequently, given the frequent movements of the dolphins across the administrative border, line-transect surveys limited to Hong Kong territorial waters only [43,47,48] can effectively sample only a portion of the population range and are given to generate lower estimates and under-represent the number of dolphins that use Hong Kong waters as part of their home range. Furthermore, annual estimates that do not adequately address seasonal fluctuations, as it is the case with the currently ongoing Hong Kong line-transect surveys [47,48], may be subject to negative bias due to the inherent seasonal variation.

Another source of error originates from the fact that the line-transect data used for the abundance estimates did not include the region of southwest Lantau Island, from Fan Lau in the west to Shek Pik peninsula and Soko Islands in the east/south-east [47,48], a region known to represent one of the limited few key areas of the dolphins' critical foraging habitat in Hong Kong waters [80]. Approximately 20% of the photo-ID database records used in our study was collected in this area, indicating its importance to the dolphins. Similarly excluded from the Hong Kong line transect surveys was the Deep Bay area in the northwest of Hong Kong territorial waters, which was historically used by the dolphins and is still so at some level ([41]; T.A. Jefferson, pers. comm.). Such arbitrary exclusion of certain areas from transect sampling and not taking them into account in the estimates of the abundance of dolphins in Hong Kong will inevitably cause bias. These obvious shortcomings of the currently ongoing line-transect monitoring programme received a stark criticism at a recent meeting of the Hong Kong environmental management authority (Marine Mammal Conservation Working Group of the Agriculture, Fisheries and Conservation Department (AFCD) of HKSAR government, 14^th^ July, 2016), and it appears that previously reported abundance estimates [e.g. 47,48] may have been underestimated by at least 20%.

A recent study by W.H. Wong and L. Karczmarski (study in progress) indicates a considerable shift in the distribution of humpback dolphins in Hong Kong, with waters off west-southwest Lantau Island (an area not included in abovementioned line-transect estimates [47,48]) becoming increasingly more important for the dolphins' daily occurrence and nutritional needs. On the contrary, waters of north Lantau, the only section of Hong Kong territorial waters under legal protection specifically for the conservation of humpback dolphins, are increasingly degraded by large-scale coastal construction projects [25,122] and are progressively less used by the dolphins [123]. Coincidentally, in contrast to our mark-recapture estimates which were gradually increasing, albeit only slightly, across the study period (as discussed earlier), the line-transect estimates by Hung [47,48] display a declining trend. There may be several reasons for the declining line-transect estimates and methodological inadequacies cannot be ruled out (for a detailed critique see Wilson et al [40]). However, one other plausible possibility is that, because of the exclusion of the southwest Lantau region from the line-transect estimates, the apparent "decline" may reflect nothing else than the inability of detecting a shift in dolphin distribution from the area covered by the reported estimates to an area that has been arbitrarily excluded from the line-transect estimates and which happens to be frequently used by the dolphins, and likely increasingly so in a near future [123]. We therefore urge that extreme caution should be exercised when interpreting the findings reported by the Hong Kong line-transect monitoring programme [47,48]. A failure to recognise its limitations may lead to misguided management decisions (for further details and discussion see [25,40,110]).

Our study indicates that humpback dolphins seen in Hong Kong waters represent part of a larger population and Hong Kong territorial waters represent only part of a considerably larger range of this population. Although the previously suggested decline in the numbers of dolphins in Hong Kong waters [47,48] may be an artefact of a methodological imperfection of earlier studies and is unlikely to reflect accurately the current trend in Hong Kong, a population decline of a considerably greater magnitude (~2.5% loss per annum) is currently underway and affects the entire PRE population [27]. Consequently, our study, with the estimates of population parameters based on mark-recapture data collected in Hong Kong waters only, may under-represent the current status of the larger PRE population and the pressure it faces [25], and instead reflect the increasing relative importance of western Hong Kong waters compared to other parts of the PRE. Although no comprehensive assessment of the multitude of anthropogenic impacts on the dolphins across the PRE has ever been undertaken, there are indications that the environmental conditions in Hong Kong waters, albeit severely degraded, are still not as deteriorated as those in other parts of the PRE across the administrative border [25]. This underscores the importance of conservation efforts and management measures that are in effect in Hong Kong as they are of considerable importance for the entire PRE population.

The conservation status of the Indo-Pacific humpback dolphin has recently been re-assessed under the IUCN categories and criteria as Vulnerable [36]; previously listed as Near Threatened [33]. The regional status of local populations, however, can differ, sometime substantially [34]. The PRE population, assessed as a distinct population unit meets the criteria for conservation status classification as Endangered, approaching Critically Endangered under the IUCN Criterion A3b [25,27]. Recent analyses suggest that the population is currently at its viability threshold [109] and further decline with the current rate of ~2.5% loss per annum [27] will take the population below the demographic threshold within the lifespan of one generation, at which point adverse consequences of demographic stochasticity may likely set in and further impair the population long-term viability [109].

As Hong Kong territorial waters represent only a small part of the PRE ecosystem and the range of dolphins seen in Hong Kong is not restricted by political borders, the relevant conservation management plan has to take a form of a cross-boundary management strategy which includes, among other, fishery regulations with effective monitoring mechanisms [120,124], strict standards and control of pollutant discharge to mitigate pollutant accumulation [23,125,126], and a network of marine protected areas that are designed based on sound ecological evidence [80] and encompass sufficient quantity and quality of critical habitats that can accommodate a sufficiently viable population [109]. As the estimated decline of the PRE dolphin population is of a considerable magnitude [27], it has likely been underway for a long time [110]. Implementing effective management is therefore urgently needed, but it is unlikely to succeed without effective monitoring strategy that can timely and accurately detect population trends and potential management pitfalls.

Huang et al [27] points out that it would take as long as a lifespan of 1–3 generations of the PRE humpback dolphins to have the recently estimated trend (~2.5% decline per annum) detected by the Hong Kong line-transect monitoring programme. During that time, however, a substantial part of the population would have been already lost. T.A. Jefferson argues (pers. comm.) that these shortcomings are not due to limitations of the line-transect technique itself but primarily a result of technical and analytical inadequacies of its application in Hong Kong over the past decade. A revision of the current monitoring strategy is therefore much needed and should be considered as a matter of urgency. This should include a scrupulous application of photo-ID mark-recapture techniques which can facilitate quantitative and timely assessments of population viability and trend, and assessment of the conservation status relative to estimated population parameters (see Karczmarski et al [109]). The study presented here, a first of its type in this region, provides methodological basis and a comprehensive database for such broad practical application in Hong Kong and across the PRE, and indicates a wealth of information that can be obtained when rigorous analytical techniques are explored to their full potential despite spatial restrictions of the study area. We believe that this study provides a good example of a model system that can be applied to each of the humpback dolphin populations known to occur along the Chinese coast as well as other coastal delphinid populations elsewhere.

## Supporting information

S1 Table Prey species of humpback dolphins in Pearl River Estuary and their annual peak of abundance in Hong Kong waters. (DOCX)
