# Supplementary material for: Indo-Pacific humpback dolphins (Sousa chinensis) in Hong Kong: Modelling demographic parameters with mark-recapture techniques
Source: PLoS One. 2017 Mar 29;12(3):e0174029. doi: 10.1371/journal.pone.0174029 (PMC5371324; doi:10.1371/journal.pone.0174029)
Supplement: S1 Table — (DOCX) [file pone.0174029.s001.docx]

**Indo-Pacific Humpback Dolphins (*Sousa chinensis*) in Hong Kong: Modelling Demographic Parameters with Mark-Recapture Techniques**

Stephen C.Y. Chan, Leszek Karczmarski*

The Swire Institute of Marine Science and School of Biological Sciences, Faculty of Science,
The University of Hong Kong, Cape d’Aguilar, Shek O, Hong Kong

**Supporting Information**

Stomach content analyses (Barros et al [1]; W. Lin, Sun Yat-sen University, unpublished) indicate that primary prey species of humpback dolphins in the Pearl River Estuary (PRE) include Belanger's croaker (*Johnius belangerii*), lion head croaker (*Collichthys lucida*), anchovies (*Thryssa* spp.) and largehead hairtail (*Trichiurus lepturus*). Most of these species peak in abundance during summer months (May – October) as shown in Table S1. Seawater salinity and temperature are thought to be the primary factors affecting abundance of many fish species in the PRE [2].

**S1 Table. Prey species of humpback dolphins in Pearl River Estuary and their annual peak of abundance in Hong Kong waters.**

| Scientific name | Common name | Peak abundance | Source |
| --- | --- | --- | --- |
| *Johnius belangerii* | Belanger's croaker | March – April | Pitcher et al [3] |
|  |  | June | ERM [4] |
|  |  | September | ERM [4] |
| *Collichthys lucida* | Lion head croaker | June | Pitcher et al [3]; |
|  |  |  | ERM [4] |
| *Thryssa setirostris* | Anchovies | May – July | AFCD HKSAR [5] |
| *Thryssa vitrirostris* | Anchovies | August – September | AFCD HKSAR [5] |
| *Thryssa dussumieri* | Anchovies | May – September | AFCD HKSAR [5] |
| *Thryssa mystax* | Anchovies | May – August | AFCD HKSAR [5] |
| *Thryssa lepturus* | Anchovies | March – June | AFCD HKSAR [5] |

**References**

1. Barros NB, Jefferson TA, Parsons ECM. Feeding habits of Indo-Pacific humpback dolphins (*Sousa chinensis*) stranded in Hong Kong. Aquat Mamm. 2004; 30: 179-188.
2. Tam YK, Ni IH, Yau C, Yan MY, Chan WS, Chan SM, et al. Tracking the changes of a fish community following a megascale reclamation and ensuing mitigation measures. ICES J Mar Sci. 2013; 70: 1206–1219.
3. Pitcher TJ, Watson R, Courtney A, Pauly D. Assessment of Hong Kong's inshore fishery resources. Fisheries Centre Research Reports, The University of British Columbia. 1998; 6: 148pp.
4. ERM. Fisheries resources and fishing operations in Hong Kong waters: final report prepared by Environmental Resources Management. Report submitted to HKSAR Agriculture and Fisheries Department. 1998; 255pp.
5. AFCD HKSAR. Hong Kong Marine Fish Database. HKSAR Agriculture, Fisheries and Conservation Department (AFCD). 2015. Available from: http://www.hk-fish.net/eng/database/index.htm.
